# Waveguide-integrated mid-infrared photodetection using graphene on a scalable chalcogenide glass platform

**DOI:** 10.1038/s41467-022-31607-7

**Published:** 2022-07-07

**Authors:** Jordan Goldstein, Hongtao Lin, Skylar Deckoff-Jones, Marek Hempel, Ang-Yu Lu, Kathleen A. Richardson, Tomás Palacios, Jing Kong, Juejun Hu, Dirk Englund

**Affiliations:** 1 Massachusetts Institute of Technology, 77 Massachusetts Avenue, Cambridge, MA 02139 USA; 2 University of Central Florida, 4000 Central Florida Boulevard, Orlando, FL 32816 USA; 3 Present Address: State Key Laboratory of Modern Optical Instrumentation, College of Information Science and Electronic Engineering, Zhejiang University, 310027 Hangzhou, China

**Keywords:** Mid-infrared photonics, Optical properties and devices

## Abstract

The development of compact and fieldable mid-infrared (mid-IR) spectroscopy devices represents a critical challenge for distributed sensing with applications from gas leak detection to environmental monitoring. Recent work has focused on mid-IR photonic integrated circuit (PIC) sensing platforms and waveguide-integrated mid-IR light sources and detectors based on semiconductors such as PbTe, black phosphorus and tellurene. However, material bandgaps and reliance on SiO_2_ substrates limit operation to wavelengths *λ* ≲ 4 μm. Here we overcome these challenges with a chalcogenide glass-on-CaF_2_ PIC architecture incorporating split-gate photothermoelectric graphene photodetectors. Our design extends operation to *λ* = 5.2 μm with a Johnson noise-limited noise-equivalent power of 1.1 nW/Hz^1/2^, no fall-off in photoresponse up to *f* = 1 MHz, and a predicted 3-dB bandwidth of *f*_3dB_ > 1 GHz. This mid-IR PIC platform readily extends to longer wavelengths and opens the door to applications from distributed gas sensing and portable dual comb spectroscopy to weather-resilient free space optical communications.

## Abstract (web summary)

Mid-infrared photonic integrated circuits (PICs) are important for sensing and optical communications, but their operational wavelengths are usually limited below 4 *μ*m. Here, the authors report the realization of photothermoelectric graphene photodetectors incorporated in a chalcogenide glass-on-CaF2 PIC operating at 5.2 *μ*m, showing promising results for gas sensing applications.

## Introduction

Mid-IR absorption spectroscopy is a critical tool for chemical sensing and analysis, especially for inert gases that evade detection by chemical reaction-based sensors. Many such gases derive their inertness from halogenated chemistries and thus exhibit global warming potential due to carbon-halogen stretching modes resonant in the thermal IR^1,2^. To facilitate sensor deployment for greenhouse gas leak detection and other chemical sensor application areas, there exists a strong need to transition from co-packaged discrete components to compact and chip-integrated sensors.

To address this challenge, mid-IR photonic integrated circuit (PIC) platforms have been investigated to reduce optical gas sensors to the size of a chip. Recent work has demonstrated integrated optical methane^3^ and volatile organic compound^4^ sensing, but required coupling to off-chip sources and detectors. However, integrating the detector on-chip is more compact and can improve sensitivity by reducing the volume of active material able to generate thermal noise. Su et al. achieved integration of a PbTe photoconductor and demonstrated methane sensing at a wavelength of *λ* = 3.31 μm^5^, but their platform is limited to *λ* ≲ 4 μm due to absorption in the SiO_2_ substrate^6^ and by PbTe’s absorption cutoff^7^. Waveguide-integrated detectors based on narrow-gap 2D materials black phosphorus^8^ and tellurene^9^ have also been demonstrated, but they too are bandgap-limited to *λ* ≲ 4 μm.

Here we exceed the wavelength limit of previous demonstrations using graphene-based detectors on an extended-transparency waveguide platform. While graphene integrated detectors have shown promise at telecom wavelengths^10^, the material’s advantages are magnified further at longer wavelengths due to the thermal nature of the photothermoelectric (PTE) response mechanism^11,12^ and due to the impact of optical plasmon scattering at short wavelengths^13^. Integrated photodetection with graphene has been demonstrated at wavelengths up to 3.8 μm^6^ and with chalcogenide glass waveguides^14^, but on SiO_2_ platforms. To access longer wavelength operation and achieve good sensitivity at zero-bias, we introduce a Ge_28_Sb_12_Se_60_ (GSSe)-on-CaF_2_ waveguide platform supporting gated PTE-based graphene photodetectors. These key changes allow us to extend operation to a wavelength of *λ* = 5.2 μm while achieving a Johnson noise-limited noise-equivalent power (NEP) of 1.1 nW/Hz^1/2^. By comparing the gate voltage maps of our device’s resistance, transmittance, and responsivity with a photothermoelectric model, we extract material quality parameters of the graphene channel, revealing a path to further reduce the device’s NEP by shrinking the optical mode size in tandem with the graphene channel.

## Results

### Device design and responsivity measurement

**Fig. 1:**
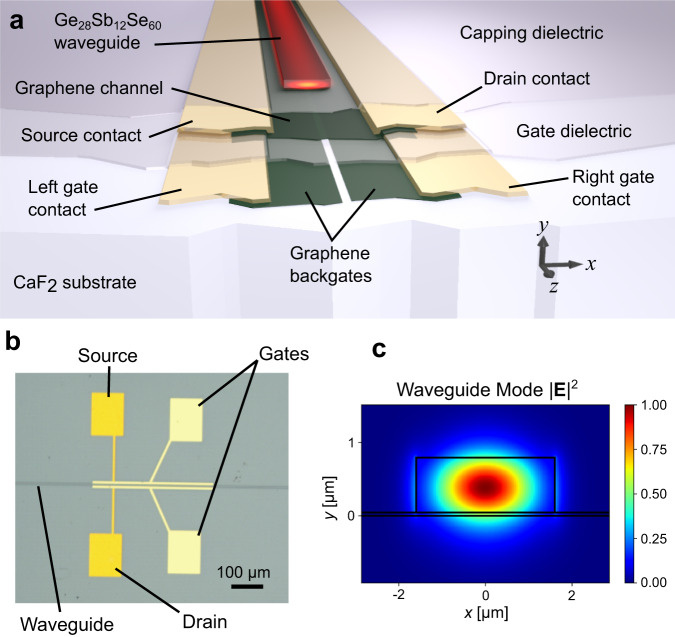
Device geometry. **a** Illustration of the device cross-section perpendicular to the waveguide axis. The optical mode supported by the GSSe waveguide evanscently couples to and is absorbed by the graphene channel, which is gated by two graphene back-gates to induce a pn-junction. **b** Optical image of the device depicting source, drain and gate contact pads. **c** Depiction of the optical guided mode at *λ* = 5.2 μm.

Figure 1a, b illustrate the platform and photodetector design. The device consists of a single-mode GSSe waveguide fabricated on top of a 5.4 μm wide by 300 μm long, CVD-grown graphene channel, flanked on either side by source and drain contacts placed far enough away from the optical mode to avoid excess loss. Beneath the graphene channel are pair of CVD graphene back-gates, separated by a 400 nm gap and used to electrostatically induce a pn-junction along the center of the channel. We use HfO_2_ as the gate dielectric and as an airtight capping layer. The device is fabricated on a CaF_2_ substrate, transparent up to *λ* = 8 μm. Figure 1c depicts the resulting waveguide mode at *λ* = 5.2 μm.

We use lock-in measurement to characterize our detectors, focusing light from a *λ* = 5.2 μm QCL source into our chip’s input facet. Light exiting the chip is focused onto an InAsSb photodetector and amplified for transmission measurement. Supplementary Fig. 1a depicts this beam-path in more detail. We operate the device under zero-bias voltage to avoid introducing electronic shot noise and to prevent channel conductivity fluctuations from manifesting as 1/*f* noise^15^. For the following low-frequency measurements we use a lock-in amplifier to measure the photovoltage directly with no preamplification.

**Fig. 2:**
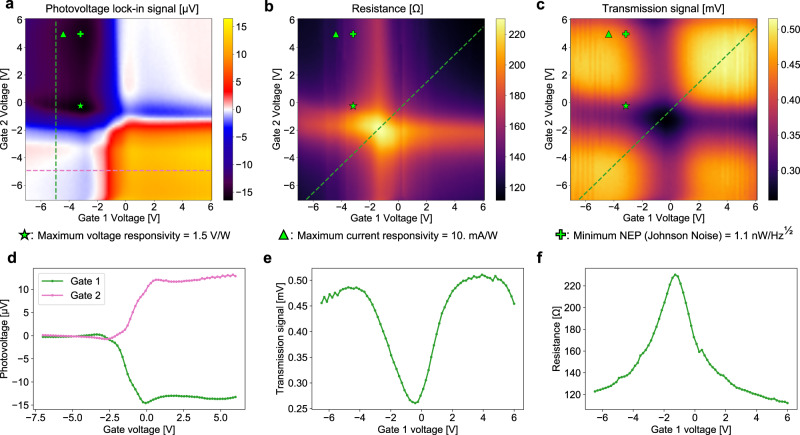
Gate voltage maps. **a** Measured zero-bias photovoltage produced by the device as a function of the two gate voltages. **b** Total device resistance as a function of the two gate voltages. **c** Lock-in signal reflecting power measured by an InAsSb photodetector at the focal point of our output facet collection lens, used to monitor transmission of the device as a function of the gate voltages. The star, triangle, and cross symbols on each gate voltage map represent the optimum operating points for maximum voltage responsivity, maximum current responsivity, and minimum NEP, respectively. The power-normalized transmittance is plotted in Supplementary Fig. 3b. **d**, **e**, **f** Plots of line sections indicated with dashed lines in panels **a**, **b**, and **c**, respectively.

Figure 2a, b, and c plot the photovoltage, resistance, and transmission lock-in signals versus both gate voltages for one such photodetector (“Device A”). Here, we modulate the *λ* = 5.2 μm QCL source at 3.78 kHz with a guided “on” power of 11 μW at the detector input. From our photovoltage and resistance maps, alongside the power and waveguide loss calibrations described in Supplementary Note 1, we infer the gate voltage pairs that optimize the voltage responsivity, current responsivity, and NEP with respect to Johnson noise, indicated with green markers in Fig. 2. For these, we arrive at 1.5 V/W, 10.  mA/W, and 1.1 nW/Hz^1/2^, respectively. The observed photovoltage gate map indicates a PTE response mechanism, evidenced by the six-fold sign change pattern around the graphene channel’s charge neutral point^11^. Figure 2d, e, and f show line slices of the voltage maps as indicated by the dashed lines in Fig. 2a, b, and c of the same color. Figure 2d, in particular, highlights the changes in slope associated with PTE-based detectors^11^.

### Photothermoelectric device model

**Fig. 3:**
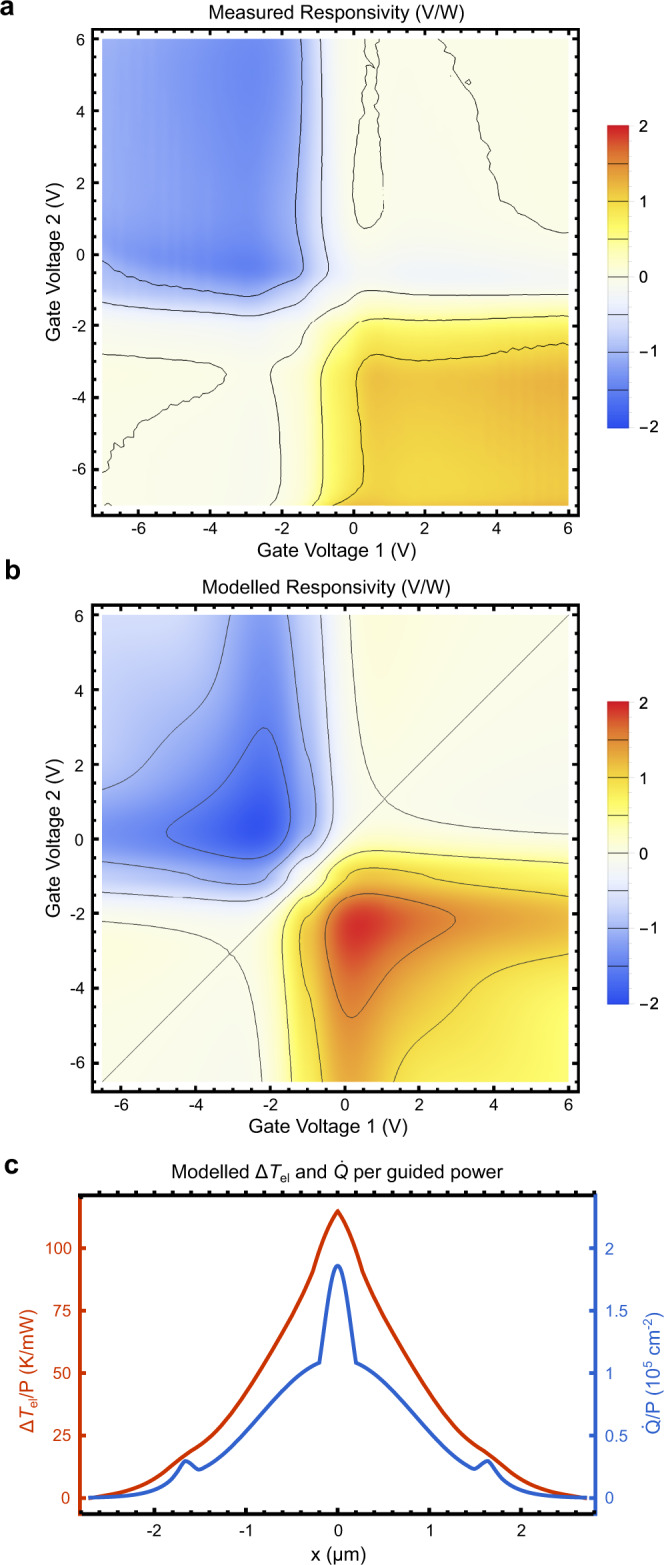
Experiment/model comparison. **a**, **b** Contour plots of the **a** measured and **b** modeled responsivity maps of our device, evaluated with *τ*_DC_ = 3.5 fs, *τ*_IR_ = 40 fs, *σ*_n_ = 2 × 10^12^ cm^−2^, *τ*_eph_ = 50 ps, and *α*_e_ = 2.5 mm^−1^. **c** Electron temperature increase Δ*T*_el_ and absorbed optical power per area $\dot{Q}$ profiles in the graphene channel per guided optical power at gate voltages of {−2.35 V,  0.35 V}, chosen to maximize the modeled photoresponse, and other parameters as above.

To confirm our understanding of device operation and elucidate the prospects for performance improvement, we apply the formalism introduced in Song et al.^12^ to calculate the electronic temperature distribution and Seebeck photovoltage in the graphene channel under illumination. Figure 3a, b compare our measured and modeled voltage responsivities using calculations described in the Methods section. The performance of our device depends on several fitting parameters, whose definitions and approximate values (derived from our measured data) we provide in Table 1. We describe our fitting process in Supplementary Note 3. Critically, all features of the modeled responsivity map in Fig. 3b up to an overall scale factor from *τ*_eph_ are established a priori from fitting parameters extracted from the device transmittance and resistance maps, with only *τ*_eph_ obtained by matching the scales of the measured and modeled responsivities. The resemblance between Fig. 3a and b thus reflects the validity of our PTE model and is not due to over-fitting. In Fig. 3c, we plot the solution to Eqn. (6), Δ*T*_el_(*x*), as well as the source term $\dot{Q}(x)$. The thermal transport model predicts that 9 μW of guided power raises the temperature of the graphene channel’s electron gas by as much as 1 K along the center of the device.

**Table 1:** Device parameters and approximate values.

*τ*_DC_	Drude scattering time at DC	≈3.5 fs
*τ*_IR_	Drude scattering time at IR	30–50 fs
*σ*_n_	Standard deviation of native carrier concentration due to spatial inhomogeneity	1.5–2.5 × 10^12^ cm^−2^
*E*_Fc_	Native Fermi level of graphene channel	≈ 0.17 eV
*E*_Fg_	Native Fermi level of graphene gates	≈ 0.48 eV
*τ*_eph_	Electron-phonon cooling time	≈ 50 ps
*α*_e_	Excess light attenuation within device	2–3 mm^−1^

### Device bandwidth and noise performance

**Fig. 4:**
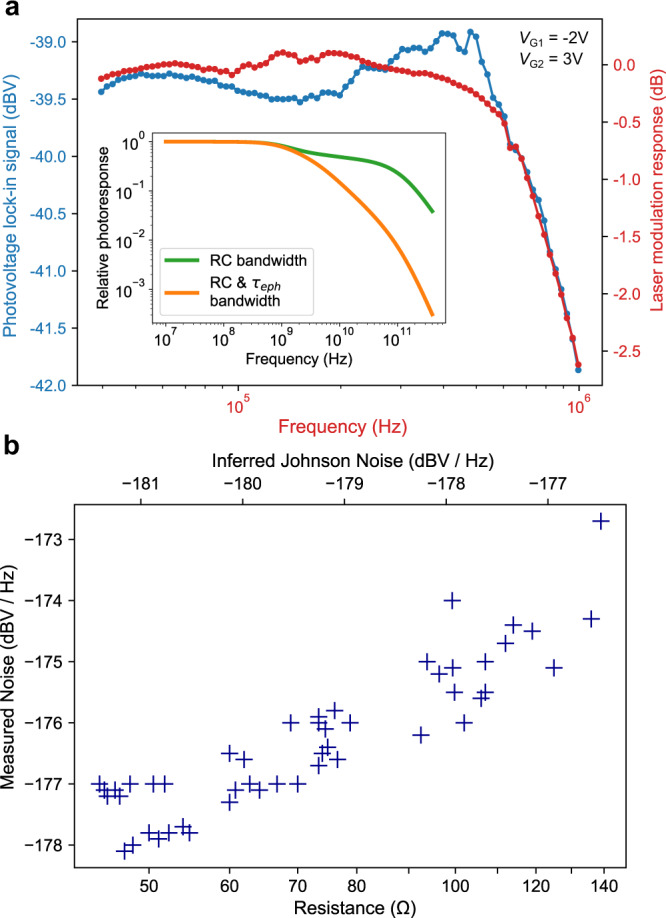
Bandwidth and noise properties. **a** Comparison of the frequency response of our photodetector with that of the laser current modulation itself. The consistency between the two indicates that the photodetector frequency response exceeds 1 MHz. Inset: Simulated GHz-range photodetector frequency response, with and without considering the impact of the electron-phonon cooling time *τ*_eph_. **b** Measured noise spectral density versus resistance and corresponding Johnson noise spectral density of Device B, without illumination, for the 49 pairs of gate voltages {*V*_g1_, *V*_g2_} where each *V*_gn_ is varied from −6 V to 6 V in steps of 2 V. Measurement was performed at *T* = 293 K.

Current modulation of our QCL source permits frequency response measurements up to its modulation bandwidth of 1 MHz. To account for the modulation response of our laser, we measure the photovoltage of Device A alongside that of a fast InAsSb photodiode. The comparison shown in Fig. 4a indicates that our device is faster than our laser’s modulation bandwidth. We thus use a COMSOL model to find the actual RC contribution to our device’s frequency response, plotted in the inset of Fig. 4a. We also plot the product of the RC-limited frequency response and the *τ*_eph_-limited frequency response with an assumed ${(1+{(2\pi {\tau }_{{{{{{{{\rm{eph}}}}}}}}}f)}^{2})}^{-0.5}$ dependence, which applies as the electron-phonon cooling length $\ell =\sqrt{\kappa {\tau }_{{{{{{{{\rm{eph}}}}}}}}}/{C}_{{{{{{{{\rm{el}}}}}}}}}}\approx 230\,{{{{{{{\rm{nm}}}}}}}}$ is narrower than our device channel^12^. We thus predict a 3-dB cutoff frequency of *f*_−3dB_ ≈ 1.3 GHz, dominated by the capacitance between the graphene back-gates.

To investigate our device’s noise performance, we modulate the QCL current at 30 kHz, amplify the photovoltage with a low-noise preamplifier and inspect using a signal analyzer. As shown in Supplementary Fig. 8, we observe in Device A no broadening of the 30 kHz photoresponse peak at offset frequencies as low as 0.1 Hz, indicating long-term responsivity stability. We then measure the un-illuminated noise spectral density and resistance versus both gate voltages. Figure 4b shows the resulting data for a Device B of identical design to Device A, organized by resistance and compared to the expected Johnson noise spectral density. We observe excellent consistency between the measured and predicted noise, with a 2 − 4 dB discrepancy consistent with the specified noise figure of our preamplifier, corroborating our earlier claim of Johnson-noise-limited NEP.

To demonstrate our device’s utility, we analyze its predicted gas-sensing performance, summarized from Supplementary Note 6. The minimum detectable gas concentration for a given waveguide platform and photodetector is given by^16^:

1 $${p}_{{{{{{{{\rm{gas,min}}}}}}}}}=e\,\frac{{\alpha }_{{{{{{{{\rm{base}}}}}}}}}\,{{{{{{{\rm{NEP}}}}}}}}}{a{n}_{{{{{{{{\rm{g}}}}}}}}}{{{\Gamma }}}_{{{{{{{{\rm{E}}}}}}}}}{I}_{0}},$$

where *I*_0_ is the source power, *α*_base_ is the waveguide attenuation coefficient in the absence of gas, *a* is the specific attenuation coefficient of the gas, *n*_g_ is the guided mode group index, Γ_E_ is the confinement factor of electric field energy within the gaseous medium, and $e=\exp (1)$. For detection of nitric oxide (NO), with an absorption peak at *λ* = 5.24 μm and a specific attenuation of approximately *a* ≈ 70 m^−1^atm^−1^ at low concentrations^17^, we arrive at ${p}_{{{{{{{{\rm{gas,min}}}}}}}}}=74\,{{{{{{{\rm{\mu }}}}}}}}{{{{{{{\rm{atm}}}}}}}}/\sqrt{{{{{{{{\rm{Hz}}}}}}}}}$ for a 1 mW illumination source. Assuming a measurement bandwidth of 0.1 Hz over which we have measured our photoresponse to be stable, we find ${p}_{{{{{{{{\rm{gas,min}}}}}}}}}=23\,{{{{{{{\rm{ppm}}}}}}}}$, roughly equal to the National Institute of Occupational Safety and Health (NIOSH) recommended exposure limit (REL) of 25 ppm^18^. Removing the slightly lossy HfO_2_ dielectric underneath the gas-light interaction waveguide could decrease ${p}_{{{{{{{{\rm{gas,min}}}}}}}}}$ considerably, as waveguide losses down to 0.7 dB/cm have been demonstrated at the same wavelength using a similar chalcogenide glass and liftoff process^19^.

## Discussion

Although our demonstration is limited to *λ* = 5.2 μm by light source availability, the optical conductivity of our graphene inferred from the fitting parameters in Table 1 remains relatively constant and even increases at longer wavelengths due to intraband absorption as shown in Supplementary Fig. 7. We thus expect our platform to scale to *λ* = 10 μm and beyond, perhaps requiring a BaF_2_ substrate for extended transparency, with little reduction in performance owing to the PTE effect’s thermal nature. In Table 2 we compare our device’s performance with various off-the-shelf detectors. Although its NEP is not yet on par with commercial options, its predicted bandwidth may be useful for dual-comb spectroscopy-based integrated gas analyzers^20^. Additionally, the vacuum requirement of VO_*x*_ bolometers may complicate co-packaging and introduce coupling losses, and the high cost of HgCdTe may preclude use in broadly deployed sensor networks.

**Table 2:** Comparison of our detector with inferred room-temperature performance metrics for two HgCdTe photodiodes optimized for two different wavelengths (from ref. ^31^) and a VO_*x*_ bolometer (from ref. ^32^) available off the shelf. For the photodiodes, the NEP is extrapolated from the specified detectivity for a detector scaled down to match the size of a diffraction-limited spot with NA = 0.3, which is the acceptance NA of these detectors. For the bolometer, we give the NEP of a single 17 × 17 μm bolometer pixel as calculated from the specified noise-equivalent temperature difference as described in Rogalski^7^.

	HgCdTe PD *λ*_opt_ = 5.0 μm	HgCdTe PD *λ*_opt_ = 10.6 μm	VO_*x*_ bolometer	This work
NEP $[{{{{{\rm{pW}}}}}}/\sqrt{{{{{\rm{Hz}}}}}}]$	1, *λ* = 5.2 μm 0.2, *λ* = 5.0 μm	10, *λ* = 5.2 μm 40, *λ* = 10.6 μm	0.9	1100
*f*_−3dB_ [MHz]	1.3	106	10 Hz	1300 (pred.)
Vacuum required?	No	No	Yes	No
Waveguide-integrated?	No	No	No	Yes

In conclusion, we have demonstrated a PTE-based graphene photodetector, integrated in a scalable chalcogenide glass waveguide platform with an NEP of 1.1 nW/Hz^1/2^ and a bandwidth exceeding *f*_−3dB_ = 1 MHz. We have modeled the bandwidth to approach 1.3 GHz and we predict similar performance at longer wavelengths for scaled-up devices enabled by the transparency of GSSe beyond *λ* = 10 μm^21^. Finally, we have shown that our device and waveguide platform would enable NO detection at concentrations comparable to its REL. Substantial improvements are likely using metal-insulator-metal^10^ or dielectric slot waveguides to concentrate the optical mode to within a cooling length of the pn-junction, which would also increase the attenuation of the guided mode and thus decrease the device footprint needed to absorb an optical signal. Gapped bilayer graphene may also be investigated as an alternative to monolayer graphene to reduce thermal noise^22^. The PIC platform further promises to support a full toolkit of mid-IR active devices including on-chip quantum cascade light sources^23^, and may even leverage the same graphene material platform for devices such as graphene modulators^14^ and hot-electron-based^24^ or gapped bilayer graphene light sources. The platform could also be adapted to alternative mid-IR waveguide approaches, such as suspended Ge, as necessary to reach longer wavelength ranges^25^. Chalcogenide glass could then supplement such a platform by enabling designs where the graphene channel is sandwiched between the Ge and high-index glass to increase overlap with the optical mode. This research represents the first foray into waveguide-integrated detectors operating beyond *λ* = 4 μm, paving the way towards 2D-material-enabled integrated mid-IR microsystems for gas sensing, spectroscopy^20^ and free-space optical communications^26^.

## Methods

### Photodetector fabrication

A continuous monolayer graphene film was grown on Cu foil (99.8%, Alfa Aesar, annealed, uncoated, item no. 46365) cut to a size of 15 × 2 cm^2^ in a 1-inch-diameter quartz tube furnace under atmospheric pressure. The furnace was heated to 1060 ^∘^C over 30 min under 500 sccm of Ar flow; afterwards, 15 sccm of H_2_ and 10 sccm of dilute CH_4_ (1% in Ar) were introduced as reducing gas and carbon source, respectively, and flowed for 4 h to ensure the continuity of the graphene film. Finally, the furnace was allowed to cool to 100 ^∘^C without modifying the gas flow before the CVD graphene was removed from the chamber. Our devices were fabricated on a 1" diameter by 1.0 mm thick (111)-cut CaF_2_ substrate (MTI Corporation, item CFc25D10C2). We first coated our substrate with a PMMA bilayer for liftoff (495 PMMA A6 followed by 950 PMMA A2), which features a slightly re-entrant sidewall profile after developing. We then performed e-beam lithography using an Elionix FLS-125 125 keV electron beam lithography system to pattern alignment marks on our substrate, followed by room-temperature development in 3:1 isopropanol:methyl isobutyl ketone for 90 s and isopropanol rinse for 120 s (“development process”), e-beam evaporation of 5 nm Ti/100 nm Au (Temescal VES2550) (“metal evaporation process”), and liftoff using a 4-h room-temperature acetone soak (“liftoff process”). To transfer the first layer of graphene, we first coated one side of the CVD graphene-on-Cu sheet with PMMA and removed the graphene from the other side using 90 s of oxygen RIE (16 sccm He and 8 sccm O_2_ at a pressure of 10 mTorr and an RF power of 100W, “oxygen RIE process”). We then etched away the Cu using a FeCl_3_-based etchant, followed by 2 DI water rinses, a 30-min clean in 5:1 DI water:HCl 37% in water to reduce metal ion contamination, and two more DI water rinses. After letting the graphene film sit overnight in the final evaporating dish of water, we scooped it out with our CaF_2_ substrate, blew N_2_ on the film to eliminate most of the trapped water, and then baked the sample at 80^∘^ for 30 min followed by 160^∘^ for 2 h (“graphene transfer process”). We then removed the PMMA from the graphene using acetone at room temperature, rinsed it in isopropanol and blew it dry (“PMMA removal process”), and baked the sample at 200 ^∘^C in N_2_ for 1 h to improve adhesion. To pattern the graphene back-gates, we spun on a layer of 950 PMMA A6, exposed the gates in the Elionix and developed using “development process”, etched away the exposed graphene using “oxygen RIE process” for 45 s, and removed the PMMA using “PMMA removal process”. We then spun on another 495 PMMA A6/950 PMMA A2 bilayer, exposed the metal contacts to the graphene gates using the Elionix FLS-125, and repeated “development process”, “metal evaporation process”, and “liftoff process”, but using a 2 nm Ti adhesion layer in the Ti/Au stack rather than 5 nm. After this, we evaporated 1.5 nm Al (Temescal VES2550) as an ALD seed layer, allowed the thin Al layer to oxidize in ambient, and deposited 300 cycles ≈ 30 nm of HfO_2_ ALD at 200 ^∘^C (Cambridge Nanotech Savannah 200). To define the graphene channel, we performed another “graphene transfer process”, “PMMA removal process”, 1 h N_2_ ambient 200 ^∘^C bake, 950 PMMA A6 spin-coating, Elionix FLS-125 exposure of graphene channel pattern, “development process”, “oxygen RIE process” for 45 s, and “PMMA removal process”. To define the channel contacts, we spin-coated another 495 PMMA A6/950 PMMA A2 bilayer, exposed the graphene channel contacts using the Elionix FLS-125, and performed another “development process”, “metal evaporation process”, and “liftoff process”, but using a 2 nm Ti adhesion layer in the Ti/Au stack rather than 5 nm. We then evaporated another 1.5 nm Al seed layer using the Temescal VES2550 and 150 cycles of HfO_2_ ALD at 200^ ∘^C using the Cambridge Nanotech Savannah 200 to protect the graphene channel. Finally, to pattern the GSSe waveguides, we coated the chip with 495 PMMA A11, used the Elionix FLS-125 to define the waveguides, and developed in room-temperature 3:1 isopropanol:methyl isobutyl ketone for 120 s followed by an isopropanol rinse for 120 s. The longer development time is mandated by the thicker resist film. We then evaporated 750 nm of Ge_28_Sb_12_Se_60_ followed by a quick liftoff in boiling acetone (~20 min), IPA rinse and N_2_ blow-dry, and cleaving of the chip to expose waveguide facets.

### Measurement conditions

The maps in Fig. 2a, b, and c were measured by sequentially measuring each data point column by column, bottom to top from left to right. SR830 lock-in amplifiers were used for all measurements. Prior to each data point collection, both gate voltages were reset to −7 V for 80 ms to reset the gate dielectric hysteresis (see Supplementary Note 2), then set to the desired gate voltages and allowed to dwell for 200 ms for the lock-in signal to stabilize. The lock-in filter was set to a 30 ms time constant with a 12 dB/octave falloff. The detector photovoltage in Fig. 2a was measured directly by the lock-in amplifier with no additional amplification. For the resistance map in Fig. 2b, we used our lock-in amplifier to bias the device with a 1 VRMS sine wave at 3.78 kHz through a 100 kΩ resistor to act as a current source and measured the voltage across the device with the lock-in. To produce the frequency response plots in Fig. 4a, we apply a sinusoid of variable frequency to the current modulation input of our QCL and measured the calibration and photoresponse signals with a SR844 RF lock-in amplifier. For the laser modulation response (indicated in red in Fig. 4a), we couple the laser light through a single-mode waveguide on our chip with no devices on it and directly measure the amplified transmission signal produced by the fast InAsSb detector on the output side of our chip. For the photovoltage signal (blue curve in Fig. 4a), we amplify the photovoltage produced by our detector by 40 dB using a preamplifier and measure this amplified signal with our lock-in. In all cases, we used a dwell time of 1.5 s, and the filter of our lock-in was set to 100 ms with a 12 dB/octave falloff. To measure the un-illuminated noise spectral density in Fig. 4b, we amplify the noise produced by the device using a 60 dB preamplifier and analyze the output on an FFT signal analyzer while controlling the gate voltages applied to the device. We choose to measure the averaged noise spectral density between 22 and 32 kHz where we find no electromagnetic interference-related spectral peaks in our lab environment. At the same time as the noise measurement, we also use a lock-in amplifier to measure the device resistance by recording the voltage across the device while biased with 1 VRMS through a 100 kΩ resistor, albeit at a higher frequency so as to not produce a signal in the noise measurement range. We use our signal analyzer’s band averaging feature to measure the noise spectral density for each data point. To produce the final plot, we manually record the resistance and noise spectral density for all gate voltage pairs from −6 V to 6 V in steps of 2 V.

### Device modeling

We use the Kubo formula reproduced here from Hanson^27^ to model graphene’s conductivity at DC and infrared frequencies (albeit with different values of the Drude scattering time *τ* for the different frequency ranges):

2 $$\sigma (\omega ,{E}_{F},\tau ,T)=\;	 \frac{j{e}^{2}(\omega -j{\tau }^{-1})}{\pi {\hslash }^{2}}\\ 	\times \left[\frac{1}{{(\omega -j{\tau }^{-1})}^{2}}\int\nolimits_{0}^{\infty }\varepsilon \left(\frac{\partial {f}_{d}(\varepsilon )}{\partial \varepsilon }-\frac{\partial {f}_{d}(-\varepsilon )}{\partial \varepsilon }\right)d\varepsilon \right.\\ 	\left.-\int\nolimits_{0}^{\infty }\frac{{f}_{d}(-\varepsilon )-{f}_{d}(\varepsilon )}{{(\omega -j{\tau }^{-1})}^{2}-4{(\varepsilon /\hslash )}^{2}}d\varepsilon \right]$$

where *e* is the elementary charge, ${f}_{d}(\varepsilon )={(\exp ((\varepsilon -{E}_{F})/{k}_{B}T)+1)}^{-1}$ is the Fermi-Dirac distribution and *k*_*B*_ is Boltzmann’s constant. As I will show below, graphene’s low-frequency conductivity *σ*_DC_ and infrared conductivity *σ*_IR_ affect various intermediate model parameters; *σ*_DC_ and *σ*_IR_ themselves depend strongly on *E*_*F*_, which features spatial variation due to the back-gates. For the graphene channel, we assume a constant *N*_c_ = *N*_0,c_  +  *e*^−1^*C*_g_*V*_g_ in the region above each gate, where *N*_c_ is the carrier concentration in the channel (positive for positive *E*_*F*_, negative for negative *E*_*F*_), *N*_0,c_ is the native carrier concentration at zero gate voltage, *C*_g_ is the capacitance per area of the gate dielectric, and *V*_g_ is the voltage applied to the gate in question (using a set of test devices, we measure *C*_g_ = 34.  fF/μm^2^ on our chip, corresponding to a back-gate dielectric constant of *K* ≈ 12; this is described in more depth in Supplementary Note 4). In the part of the graphene channel above the gap between the two gates, we assume a linear slope between *N*_c,1_ and *N*_c,2_. For the gates, *N*_g_ = *N*_0,g_  −  *e*^−1^*C*_g_*V*_g_, with *N*_g_ and *N*_0,g_ defined similarly to *N*_c_ and *N*_0,c_. In general, the graphene’s Fermi level and carrier concentration are related by ${E}_{F}=\hslash {v}_{{{{{{{{\rm{gr}}}}}}}}}\sqrt{\pi | N| }\,{{{{{{{\rm{sign}}}}}}}}(N)$, where *v*_gr_ is graphene’s Fermi velocity. To incorporate the blurring of the graphene’s Fermi level-dependent properties due to spatial carrier concentration variations, we convolve the Kubo formula with a Gaussian as follows:

3 $${\sigma }_{{{{{{{{\rm{DC}}}}}}}}}(N)=\frac{1}{{\sigma }_{{{{{{{{\rm{n}}}}}}}}}\sqrt{2\pi }}\int\nolimits_{-\infty }^{\infty }{e}^{-\frac{1}{2}\frac{{(n-N)}^{2}}{{\sigma }_{{{{{{{{\rm{n}}}}}}}}}^{2}}}\sigma (0,{E}_{F}(N),{\tau }_{DC},{T}_{0})\,dn$$

and similarly for *σ*_IR_(*N*) using *ω* = 2*πc*/*λ* instead of 0 and *τ*_IR_ instead of *τ*_DC_. Finally, we have $R={\sigma }_{{{{{{{{\rm{DC}}}}}}}}}^{-1}$, $\kappa ={\pi }^{2}{k}_{B}^{2}{T}_{0}{\sigma }_{{{{{{{{\rm{DC}}}}}}}}}/3{e}^{2}$ via the Wiedemann-Franz law, and $S=-d(\log {\sigma }_{{{{{{{{\rm{DC}}}}}}}}})/d{E}_{F}$^28^. *C*_el_ is obtained by convolving the heat capacity of pristine graphene with a Gaussian of standard deviation *σ*_*N*_ as in Eqn. (3), where the pristine heat capacity is given by^28,29^:

4 $${C}_{{{{{{{{\rm{el}}}}}}}}}(N){| }_{{\sigma }_{{{{{{{{\rm{n}}}}}}}}} = 0}=\int\nolimits_{-\infty }^{\infty }\varepsilon \,\frac{2| \varepsilon | }{\pi {\hslash }^{2}{v}_{{{{{{{{\rm{gr}}}}}}}}}^{2}}\frac{\partial {f}_{d}(\varepsilon -{E}_{F}(N))}{\partial T}\,d\varepsilon .$$

We use a waveguide eigenmode solver to find the mode profile of our waveguide at *λ* = 5.2 μm, using refractive indices of 1.4, 2.6, and 1.88 for the CaF_2_, GSSe, and HfO_2_, respectively. The resulting mode profile enters into our expression for ${\dot{Q}}_{{{{{{{{\rm{el}}}}}}}}}$ as follows^30^:

5 $${\dot{Q}}_{{{{{{{{\rm{el}}}}}}}}}=P\,\frac{\left(| {E}_{x}(x,{y}_{{{{{{{{\rm{c}}}}}}}}}){| }^{2}+| {E}_{y}(x,{y}_{{{{{{{{\rm{c}}}}}}}}}){| }^{2}\right){\sigma }_{{{{{{{{\rm{IR,c}}}}}}}}}(x)}{{\iint }_{{{\mathbb{R}}}^{2}}{{{{{{{\rm{Re}}}}}}}}({{{{{{{\bf{E}}}}}}}}\times {{{{{{{{\bf{H}}}}}}}}}^{* })\cdot \hat{{{{{{{{\bf{z}}}}}}}}}\,dx\,dy}.$$

Here, *y*_c_ is the *y*-coordinate of the graphene channel, and *y*_g_ would be the *y*-coordinate of the graphene gates. We may then write ${\alpha }_{{{{{{{{\rm{c}}}}}}}}}={P}^{-1}\,\int\nolimits_{-W/2}^{W/2}{\dot{Q}}_{{{{{{{{\rm{el}}}}}}}}}(x)\,dx$. Similar expressions hold for *α*_g_ in terms of *σ*_IR,g_(*x*), noting of course that *σ*_IR,g_(*x*) = 0 for *x* within the gap between the gates where there is no graphene. Finally, ${\rho }_{{{\Omega }}}=\int\nolimits_{-W/2}^{W/2}R(x)\,dx$.

Having thus obtained expressions for *κ*(*x*), *C*_el_(*x*), ${\dot{Q}}_{{{{{{{{\rm{el}}}}}}}}}(x)$, *S*(*x*), Π(*x*), *α*_c_, *α*_g_ and *ρ*_Ω_ as a function of the gate voltages, as well as *τ*_DC_, *τ*_IR_, *σ*_n_, *E*_Fc_, *E*_Fg_, *τ*_eph_, *α*_e_, and *ρ*_c_, we then solve for the increase in electronic temperature per guided power Δ*T*_el_(*x*)/*P* = (*T*_el_(*x*) − *T*_0_)/*P* using the equation:

6 $$-\frac{d}{dx}\left(\kappa \,\frac{d\,{{\Delta }}{T}_{{{{{{{{\rm{el}}}}}}}}}}{dx}\right)+{\tau }_{{{{{{{{\rm{eph}}}}}}}}}^{-1}{C}_{{{{{{{{\rm{el}}}}}}}}}{{\Delta }}{T}_{{{{{{{{\rm{el}}}}}}}}}=\eta \,{\dot{Q}}_{{{{{{{{\rm{el}}}}}}}}}-{J}_{x}\frac{d{{\Pi }}}{dx},$$

where *κ* is the 2D electronic thermal conductivity of the graphene, *τ*_eph_ is the electron-phonon cooling time, ${\dot{Q}}_{{{{{{{{\rm{el}}}}}}}}}$ is the absorbed optical power per area, *η* is the conversion efficiency of absorbed optical power to electronic heat after initial electron-phonon scattering^12^, *J*_*x*_ is the line current density in the *x*-direction, and Π is the Peltier coefficient. We are approximating the electric field to run exclusively in the *x*-direction, valid for sufficiently gradual light absorption. We assume *η* = 1, as has been previously reported in pump-probe experiments at this wavelength range^13^. The thermal electromotive force (EMF) arising from the Seebeck effect is then given by:

7 $${{{{{{{{\mathscr{E}}}}}}}}}_{x}=-\int\nolimits_{-W/2}^{W/2}S\,\frac{d\,{{\Delta }}{T}_{{{{{{{{\rm{el}}}}}}}}}}{dx}\,dx,$$

where *W* = 5.4 μm is the channel width and *S* is the Seebeck coefficient. In Eqns. (6) and (7), *κ*, *C*_el_, *S*, and Π = *ST*_el_ ≈ *ST*_0_ (for small Δ*T*_el_) are all dependent on the local Fermi level *E*_F_ of the graphene, and thus have a gate-tunable *x*-dependence, which we account for in our calculations. Combining the equations, the $\eta {\dot{Q}}_{{{{{{{{\rm{el}}}}}}}}}$ source term in Eqn. (6) gives rise to a proportional photo-induced EMF, whereas the Peltier term ${J}_{x}\frac{d{{\Pi }}}{dx}$ gives rises to a current-dependent EMF, which appears as a resistance in series with the Ohmic and contact resistances of the channel. We can thus write:

8 $$V={\overline{{{{{{{{\mathcal{R}}}}}}}}}}_{v}\,{\alpha }_{{{{{{{{\rm{c}}}}}}}}}\,P(z)-\left({\rho }_{{{\Omega }}}+{\rho }_{{{\Pi }}}+{\rho }_{{{{{{{{\rm{c}}}}}}}}}\right){J}_{x}(z)$$

where *V* is the voltage across the contacts, ${\overline{{{{{{{{\mathcal{R}}}}}}}}}}_{v}$ is the photovoltage per absorbed power per length of a cross-sectional slice of the device (i.e., dimensions of V/(W/m)), *α*_c_ is the component of the waveguide power attenuation coefficient arising from absorption in the graphene channel, *P*(*z*) is the guided power at a position along the waveguide, and *ρ*_Ω_, *ρ*_Π_, *ρ*_c_ are the Ohmic, Peltier, and contact line resistivities (dimensions of Ω ⋅ m), respectively. Averaging over *z* along the length of the waveguide we obtain:

9 $$V=\frac{{\overline{{{{{{{{\mathcal{R}}}}}}}}}}_{v}\,{\alpha }_{{{{{{{{\rm{c}}}}}}}}}}{L\,{\alpha }_{{{{{{{{\rm{tot}}}}}}}}}}\left(1-{e}^{-{\alpha }_{{{{{{{{\rm{tot}}}}}}}}}L}\right){P}_{{{{{{{{\rm{in}}}}}}}}}-\left({R}_{{{\Omega }}}+{R}_{{{\Pi }}}+{R}_{{{{{{{{\rm{c}}}}}}}}}\right)I,$$

where *I* is the current produced by the photodetector, thus describing a Thévenin equivalent source. Here, *α*_tot_ = *α*_c_ + *α*_g_ + *α*_e_ is the total guided power attenuation coefficient within the detector, including contributions not only from the graphene channel but also from the graphene gates (*α*_g_), as well as a gate-independent excess loss *α*_e_ associated with scattering and absorption from organic or metallic impurities attached to or trapped underneath the graphene sheets. Thus, the total device resistance is equal to *R* = *R*_Ω_ + *R*_Π_ + *R*_c_, and the voltage responsivity is given by:

10 $${{{{{{{{\mathcal{R}}}}}}}}}_{v}=\frac{{\overline{{{{{{{{\mathcal{R}}}}}}}}}}_{v}\,{\alpha }_{{{{{{{{\rm{c}}}}}}}}}}{L\,{\alpha }_{{{{{{{{\rm{tot}}}}}}}}}}\left(1-{e}^{-{\alpha }_{{{{{{{{\rm{tot}}}}}}}}}L}\right),$$

which we plot versus both gate voltages in Fig. 3b for the best-fit device parameters given in Table 1 obtained as described in Supplementary Note 3. All calculations are carried out in Mathematica.

## Supplementary information

Supplementary Information

## Data Availability

The datasets generated during and/or analyzed during the current study are available in the FigShare repository at 10.6084/m9.figshare.c.5514759.v1.

## Code availability

The Mathematica document used to simulate photodetector performance metrics is available in the FigShare repository at 10.6084/m9.figshare.c.5514759.v1.
