# Supplementary material for: Waveguide-integrated mid-infrared photodetection using graphene on a scalable chalcogenide glass platform
Source: Nat Commun. 2022 Jul 7;13:3915. doi: 10.1038/s41467-022-31607-7 (PMC9262905; doi:10.1038/s41467-022-31607-7)
Supplement: Supplementary file 1 — Supplementary Information [file 41467_2022_31607_MOESM1_ESM.pdf]

# Waveguide-Integrated Mid-Infrared Photodetection using Graphene on a Scalable Chalcogenide Glass Platform: Supplementary Information

Jordan A. Goldstein, Hongtao Lin, Skylar Deckoff-Jones, Marek Hempel, Ang-Yu Lu, Kathleen Richardson, Tomás Palacios, Jing Kong, Juejun Hu and Dirk R. Englund

June 5, 2022

## Supplementary Note 1. Optical Setup, Alignment, and Power and Loss Calibration

Supplementary Fig. 1a depicts the optical beam path during all optical and optoelectronic measurements. Chopped, collimated illumination at a wavelength of $\lambda = 5.2\,\mu\text{m}$ is coupled into a cleaved waveguide facet at the edge of the chip using a molded aspheric focusing lens to achieve a diffraction-limited spot. The waveguides containing our photodetectors and test devices as well as the kickback waveguides for loss measurement are designed to exit the chip at a 5 mm offset with respect to the input to reduce the amount of stray light picked up by the collection optics. The collection optics consists of a 1" high-NA germanium asphere which images the output facet at the center of an iris, behind which we place an InAsSb photodiode that monitors the out-coupled power. To align the setup, we flip the Ge collection lens, iris and photodiode out of output the beam path and use a long focal length $CaF_2$ lens and liquid nitrogen-cooled InAsSb camera to image the output facet of the chip while adjusting the chip position to achieve coupling first through a straight multimode waveguide (not shown) followed the desired S-shaped device or kickback waveguide. We then replace the long focal length lens with the high-NA germanium collection lens, flip the iris back into the collection beam path in the "open" position, and place the camera behind the iris. By adjusting the position of the collection lens to focus the out-coupled light at the center of the iris while gradually reducing the iris aperture size and monitoring the focal point on the camera, we are able to localize the focus of the collection lens at the center of the iris. Finally, we replace the camera with the InAsSb photodiode and adjust the in-coupling and out-coupling optics to maximize the signal measured by the photodiode.

To determine the loss of our waveguides and predict the optical power immediately incident upon our graphene photodetector during characterization, we have fabricated a set of five waveguide "kickback" structures of varying length, the layout of which is shown in the inset of Supplementary Fig. 1b. A large radius of $75\,\mu\text{m}$ is used for the waveguide turns to reduce the associated light leakage to negligible levels. We individually align and optimize the coupling for each of these waveguides and record the lock-in signal of the collection photodiode; these data are plotted versus the total kickback waveguide length in Supplementary Fig. 1b. We could not collect a data point for the longest kickback because we found the out-coupled light to be indistinguishable from stray illumination. Fitting the data to a decaying exponential reveals a waveguide loss of $1.1\,\text{dB/mm}$ and an in-coupled power corresponding to a lock-in signal of $46.2\,\text{mV}$. To obtain the actual in-coupled optical power, we must divide this value by the product of the voltage responsivity of the photodiode

and the efficiency of the collection optics. For the former, we obtain a value of $1.76 \times 10^3$ V/W from the manufacturer-provided calibration data. For the latter, we perform a full-wave electromagnetic simulation of the waveguide facet and extract the far-field profile of out-coupled light. Integrating the simulated optical power falling within the entrance pupil of the collection optics, we obtain a collection efficiency of 0.45. Combining the above three figures, we arrive at 58. mW coupled into the waveguide facet at optimal alignment. Accounting for waveguide loss, we obtain a power of 11. mW immediately incident upon our measured photodetector, from which we can thus calculate the responsivity figures reported in the main article.

## Supplementary Note 2. Hysteresis effect

In addition to photodetector devices, we also fabricated gated graphene test devices as illustrated in Supplementary Fig. 2a. These are similar to the detector devices described in the main article, except with only a single graphene sheet and a single contact on each graphene layer. Indeed, the detector devices can be made to behave similarly by applying the same voltage to each of the back-gates, but we report the test devices to illustrate the gate hysteresis effect observed for all devices. Supplementary Fig. 2b shows the transmission response versus applied voltage for one of our test devices. Applying a zigzag voltage sweep reveals an undesirable hysteresis pattern showing distinct curves for rising and falling voltages sweeps, which we label red and blue respectively. The same effect is observed in our photodetector devices as well. Hysteresis has been widely reported for as-deposited $HfO_2$ gates, and is attributed to trapped charge carriers within the dielectric[1, 2]. To prevent the effects of hysteresis from appearing in the gate sweeps presented in Main Fig. 2, we "reset" both gate voltages to 6.5 V prior to collecting each data point.

## Supplementary Note 3. Measured versus modelled resistance and transmittance and parameter extraction

Supplementary Fig. 3 compares the measured and modelled resistance and transmittance gate maps obtained using the mean graphene quality and waveguide loss parameters listed in Main Table 1; namely, $\tau_{\mathrm{DC}} = 3.5\,\mathrm{fs}$, $\tau_{\mathrm{IR}} = 40\,\mathrm{fs}$, $\sigma_{\mathrm{n}} = 2{\times}10^{12}\,\mathrm{cm}^{-2}$, and $\alpha_{\mathrm{e}} = 2.5\,\mathrm{mm}^{-1}$. We determined the values of these parameters to achieve the best fit simultaneously between both pairs of maps. Generally speaking, $\tau_{\mathrm{DC}}$ is inversely proportional to $R_\Omega$, $\tau_{\mathrm{IR}}$ affects the scale and modulation contrast of the transmittance $\mathcal{T} = e^{-\alpha_{\mathrm{tot}} L}$, and $\sigma_N$ affects the sharpness (width at half-max) and contrast of both. First, we infer $E_{\mathrm{Fc}}$ simply from from the gate voltage of the charge neutral point (peak in the case of $R_\Omega$ and dip in the case of $T$). Since the sharpnesses of both $R$ and $\mathcal{T}$ are largely determined by $\sigma_N$ for relatively high $\sigma_N$ as is the case for our devices, we then determine $\sigma_N$ to best match both maps. In particular, we find that the resistance map is best fit by $\sigma_{\mathrm{n}} = 1.5 \times 10^{12}\,\mathrm{cm}^{-2}$ and the transmittance map by $\sigma_{\mathrm{n}} = 2.5 \times 10^{12}\,\mathrm{cm}^{-2}$, from which we obtain the error margins quoted for $\sigma_{\mathrm{n}}$ in Main Table 1; $\sigma_{\mathrm{n}} = 2 \times 10^{12}\,\mathrm{cm}^{-2}$ represents a compromise between these two values. We then determine $\tau_{\mathrm{DC}}$ to roughly match the scale of $R$, but we allow the modelled resistance to be on the order of $10\,\Omega$ less the measured resistance to take into account the possibility of a contact resistance in this range as justified in Supplementary Section Supplementary Note 5.. The uncertainty in the actual contact resistance, as well as the imperfect fit, both contribute to uncertainty in the actual value of $\tau_{\mathrm{DC}}$. We determine $\tau_{\mathrm{IR}}$ and $E_{\mathrm{Fg}}$ to best match the measured transmittance map, and the values quoted in Main Table 1 represent our best attempt to simultaneously reflect multiple features of this map; namely, the modulation contrast between the center and corners of the map, the contrast between the upper right and other corners, and the falloff at the low-voltage edges

of the map (where intraband absorption of the graphene gates is strongest) and the high-voltage edges of the map (where interband absorption of the graphene gates is strongest). The quoted error margins of $\tau_{\mathrm{IR}}$ reflect the range over which these different features of the transmittance map are best rendered in our model, and the error margins of $\alpha_{\mathrm{e}}$ reflect the range required to match the scale of the measured transmittance map over the error range of $\tau_{\mathrm{IR}}$. We finally use these six parameters to predict the relative gate dependence of the voltage responsivity. In this way, only the overall scale factor of the responsivity is subject to a fitting parameter (namely, $\tau_{\mathrm{eph}}$); the contour of the responsivity map is, in our device's performance regime, purely predicted from parameters extracted from the resistance and transmittance maps. Therefore, the resemblance between the measured and modelled responsivity maps is not the result of tweaking parameters to achieve a fit, but rather reflects the accuracy of the photoresponse mechanism model itself using model parameters "fed forward" from the resistance and transmittance maps.

## Supplementary Note 4. Measurement of gate capacitance

We used Transmission Line Method (TLM) devices to measure both the gate capacitance and contact resistance of our devices. Shown in Supplementary Fig. 4, these devices consist of a large, contacted graphene back gate, gating a set of graphene FETs of increasing channel lengths, ranging from 5 µm to 65 µm. To extract the capacitance per area of our hafnia dielectric, we measure the capacitance between the lower graphene gate and the upper graphene/gold structure, and divide by the overlap area of these two structures. To measure the capacitance, we use a lock-in amplifier to apply a 0.5 VRMS sinusoid of variable frequency to the rightmost two metal contacts in Supplementary Fig. 4, and in the current return path we place a 10 kΩ shunt resistor, the voltage across which we monitor with the lock-in amplifier. At each frequency and for each TLM that we measure, we perform a measurement with both pads contacted and with only one pad contacted to compensate for any stray capacitance in our setup. We then subtract the measured capacitances in the "connected" and "disconnected" cases to obtain the actual device capacitance as a function of frequency, which we plot in Supplementary Fig. 5. Excluding the orange curve as an outlier, we measure a capacitance of $C \approx 30\,\mathrm{pF}$ corresponding to a capacitance per area of $C_{\mathrm{g}} = 3.4\,\mathrm{fF/\mu m^2}$ and a dielectric constant of $K \approx 12$.

## Supplementary Note 5. Measurement of TLM structure for gate resistance extraction

In additional to capacitance measurement, we also use the TLM structures for their intended purpose of evaluating the resistance of our graphene-metal contacts. For each of the six channels in each of the five TLM devices, we measure the channel resistance as a function of gate voltage using an upward voltage sweep each time to compensate for the hysteresis discussed in Supplementary Note 2. Since the gate voltage of the charge neutral point may shift slightly between measurements due to trapped charges in the gate, we then shift the measured resistance curves so that their peaks overlap. Finally, for each measured voltage offset from the Dirac peak and for each TLM, we fit the data of resistance versus channel length to a line and plot the resulting y-intercept as a function of voltage offset, manually eliminating any gate voltage sweeps showing malformed (for instanced, flattened or bimodal) resistance peaks. The resulting y-intercept curves are shown in Supplementary Fig. 6. Unfortunately, we find highly inconsistent intercept resistances between the five TLM devices, with the intercept even going negative in several cases. Therefore, we are unable to draw a quantitative conclusion regarding the contact resistance of our devices. We can at least,

however, estimate the total contact resistance (summing over both contacts) for our TLM devices to be generally in the $\approx 1 \times 10^2\,\Omega$ range; therefore, since the TLM channels are 40 μm wide, we would expect the total contact resistance of our actual photodetectors to be in the $\approx 1 \times 10^1\,\Omega$ range, which is an order of magnitude lower than our measured resistances; therefore we conclude that it can be safely ignored in our modelling as other sources of error (such as the imperfect fit between our measured and modelled resistance and transmittance maps) are much more likely to dominate the uncertainty in our analysis.

## Supplementary Note 6. Optical mode attenuation due to gas absorption and gas sensitivity analysis

Our gas sensitivity analysis follows that of Siebert at el.[3], which considers a light source at the gas absorption peak illuminating a long gas-light interaction waveguide, partially cladded with the ambient air in which gas's presence is suspected, and terminated in a noisy photodetector. However, the authors do not give a rigorous analysis of the attenuation coefficient of guided light due to the absorbing gas, which we supply here. The power attenuation coefficient in a waveguide with a perturbative source of absorption is given by [4]:

$$\alpha_{\text{wg}} = \omega_0 \frac{\iint_{\mathbb{R}^2} 2n\kappa\,|\mathbf{E}|^2\,dx\,dy}{\iint_{\mathbb{R}^2} \text{Re}(\mathbf{E}\times\mathbf{H}^*)\cdot\hat{\mathbf{z}}\,dx\,dy}, \tag{1}$$

where the complex refractive index $\bar{n} \equiv n - j\kappa$. For a partial pressure of target gas $p_{\text{gas}}$, the free space attenuation coefficient is:

$$\alpha_{\text{free-space}} = a p_{\text{gas}} = 2\kappa\omega_0/c, \tag{2}$$

therefore $\kappa = a p_{\text{gas}} c/(2\omega_0)$, and thus

$$\alpha_{\text{gas}} = a p_{\text{gas}} c \frac{\iint_{\text{gas}} n_0\,|\mathbf{E}|^2\,dx\,dy}{\iint_{\mathbb{R}^2} \text{Re}(\mathbf{E}\times\mathbf{H}^*)\cdot\hat{\mathbf{z}}\,dx\,dy}, \tag{3}$$

where $n_0 \approx 1$ is the refractive index of the gaseous medium, as $\kappa$ is only nonzero in the gaseous region of the waveguide cross-section. Since our waveguide mode is absorbed only very weakly, we now reference the expressions for group velocity $v_{\text{g}}$ and propagation constant $\beta$, respectively, given in Snyder and Love for non-absorbing waveguides[4]:

$$v_{\text{g}} = \frac{c^2\beta}{\omega_0} \frac{\iint_{\mathbb{R}^2} \text{Re}(\mathbf{E}\times\mathbf{H}^*)\cdot\hat{\mathbf{z}}\,dx\,dy}{\iint_{\mathbb{R}^2} n^2\,\text{Re}(\mathbf{E}\times\mathbf{H}^*)\,dx\,dy}, \tag{4}$$

and

$$\beta = \frac{\omega_0}{c^2} \frac{\iint_{\mathbb{R}^2} n^2\,\text{Re}(\mathbf{E}\times\mathbf{H}^*)\,dx\,dy}{\iint_{\mathbb{R}^2} n^2\,|\mathbf{E}|^2\,dx\,dy}. \tag{5}$$

Combining these two equations, the group index $n_{\text{g}}$ is thus:

$$n_{\text{g}} = \frac{c}{v_{\text{g}}} = c \frac{\iint_{\mathbb{R}^2} n^2\,|\mathbf{E}|^2\,dx\,dy}{\iint_{\mathbb{R}^2} \text{Re}(\mathbf{E}\times\mathbf{H}^*)\cdot\hat{\mathbf{z}}\,dx\,dy}. \tag{6}$$

By comparison with Eqn. 3, we finally arrive at:

$$\alpha_{\text{gas}} = a p_{\text{gas}}\, n_g \frac{\iint_{\text{gas}} n_0\,|\mathbf{E}|^2\,dx\,dy}{\iint_{\mathbb{R}^2} n^2\,|\mathbf{E}|^2\,dx\,dy} = a n_g n_0^{-1} \Gamma_{\text{E}}\, p_{\text{gas}}, \tag{7}$$

where $\Gamma_\mathrm{E}$ is the electric field confinement factor:

$$\Gamma_\mathrm{E} \equiv \frac{\iint_\mathrm{gas} n_0^2 \, |\mathbf{E}|^2 \, dx \, dy}{\iint_{\mathbb{R}^2} n^2 \, |\mathbf{E}|^2 \, dx \, dy}. \tag{8}$$

We emphasize that this is not, in general, equal to the "traditional" confinement factor $\Gamma$ defined based on the proportion of modal Poynting vector in gain/loss region, as has been previously noted[5].

The remainder of our gas sensitivity analysis follows that of Siebert et al.[3], in which an ideal gas-light interaction wavelength is found as a function of the gas concentration where maximum sensitivity to deviations is desired. For gas detection applications, maximum sensitivity at zero concentration is desired, and the ideal interaction waveguide length is simply found to be $l_\mathrm{opt} = \alpha_\mathrm{base}^{-1}$. This then leads directly to Main Eqn. 1. We emphasize that this is an idealized analysis that neglects the effects of nonidealites such as gas adsorption and desorption, interference from humidity, and laser relative intensity noise, all of which may affect the actual noise equivalent gas concentration of an actual IR absorption-based gas monitor.

## Supplementary Note 7. Optical absorption of graphene for longer wavelengths

In Supplementary Figure 8, we apply Main Eqn. 3 to predict how the real part of our graphene's optical conductivity (and thus optical absorption) would change at longer wavelengths using carrier concentration spread and Drude scattering time material parameters extracted from our device model. We find that the optical absorption remains roughly the same if not even higher at longer wavelengths, which is due to increased intraband absorption; therefore we anticipate similar device performance can be achieved at these wavelengths.

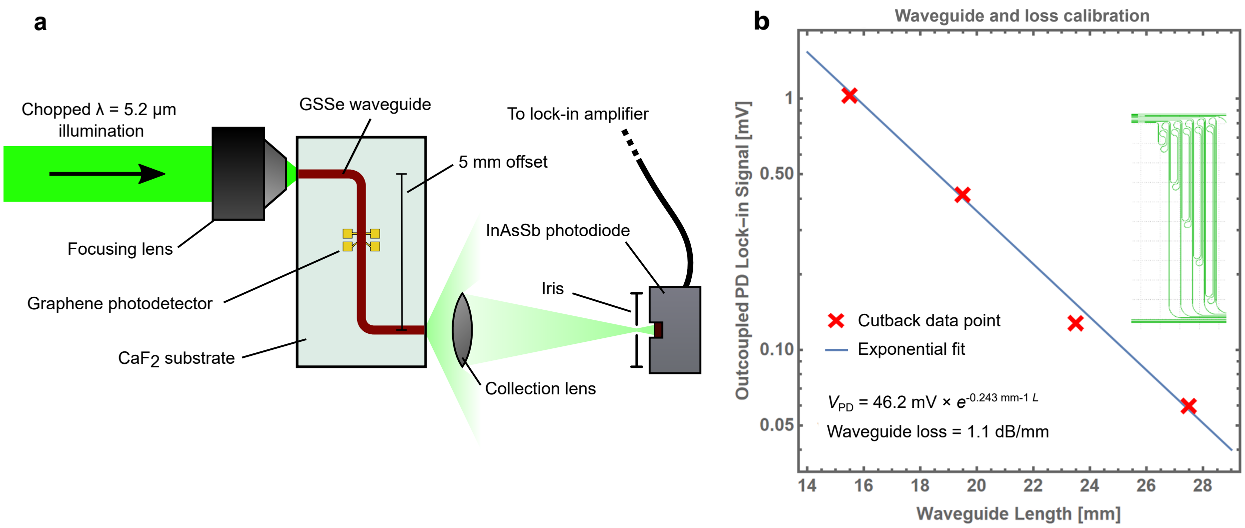


**Supplementary Figure 1: Characterization setup and loss calibration.** **a** Depiction of the in- and out-coupling beam path for optical and optoelectronic measurements. Lengths are not to scale; the chip and waveguide are magnified for clarity. **b** Output photodiode signal versus waveguide length for four waveguide kickback structures, data points represented by red crosses. The decaying exponential fit, represented by the blue line, reveals the waveguide loss and the in-coupled power, subject to a conversion efficiency accounting for the collection efficiency of the collection optics and the voltage responsivity of the photodiode. Inset: Layout of the kickback structures.

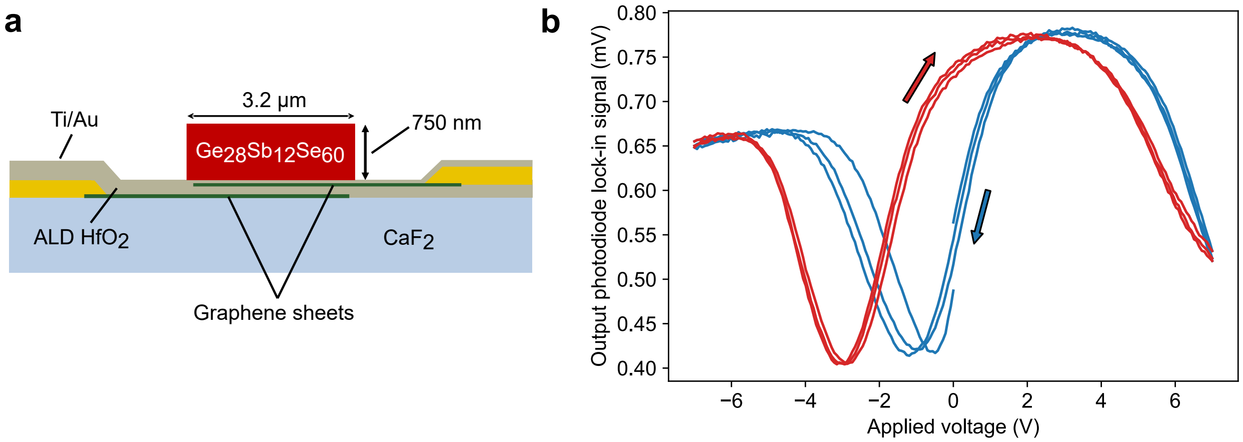


**Supplementary Figure 2: Test device cross-section and hysteresis.** **a** Illustration of the test device cross-section perpendicular to the waveguide axis. **b** Transmission versus applied voltage for one of our test devices using a zigzag voltage sweep to reveal hysteresis. Sweep direction is color-coded and indicated with arrows.

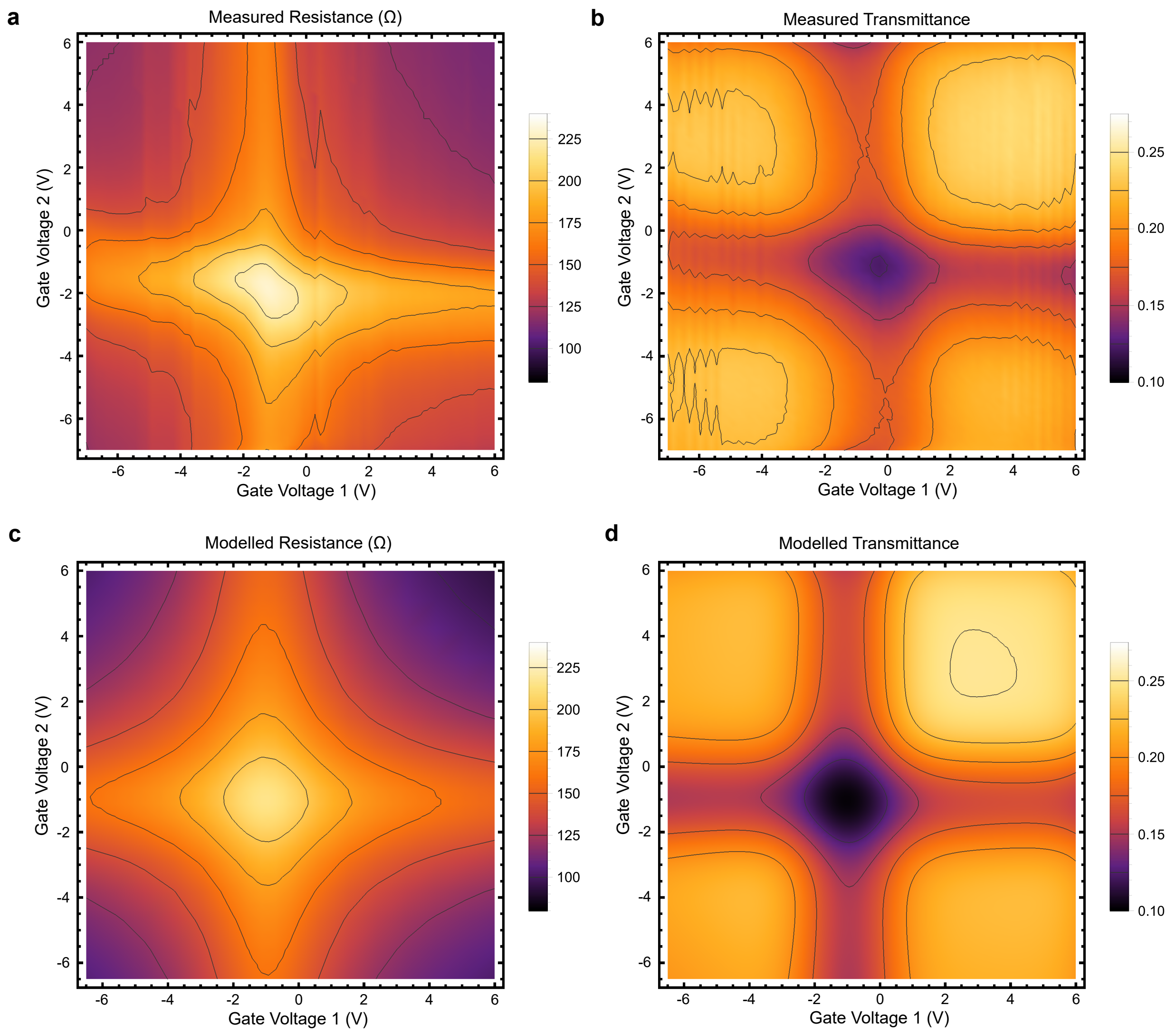


**Supplementary Figure 3: Gate voltage maps for parameter extraction. a**, **b** Contour plots of the measured resistance and transmittance maps as a function of gate voltages. **c**, **d** Contour plots of the modelled resistance and transmittance maps as a function of gate voltages using the graphene quality and waveguide loss parameters listed in Main Table 1.

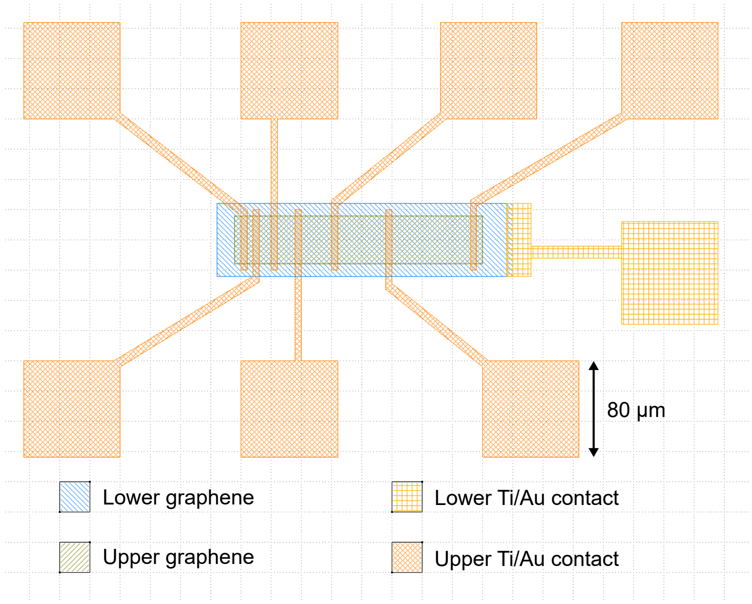


**Supplementary Figure 4: Gate capacitance and contact resistance extraction.** Transmission line method (TLM) device used for gate capacitance and contact resistance measurements.

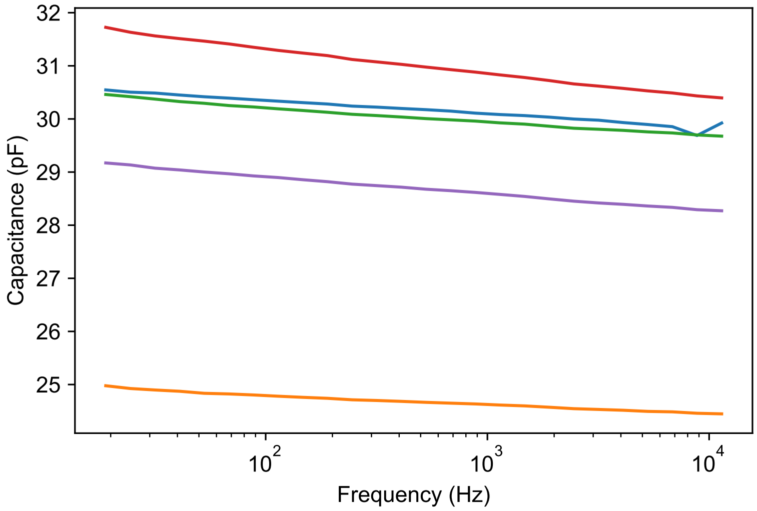


**Supplementary Figure 5: Gate capacitance data.** Capacitance versus measurement frequency for five of the TLM devices depicted in Supplementary Fig. 4.

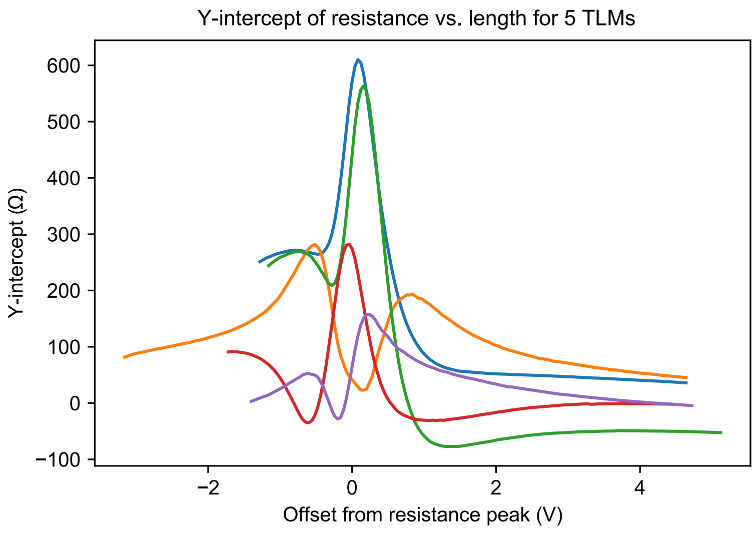


**Supplementary Figure 6: Contact resistance Y-intercepts.** Y-intercepts of resistance versus channel length as a function of gate voltage offset from the resistance peak for our five TLM devices.

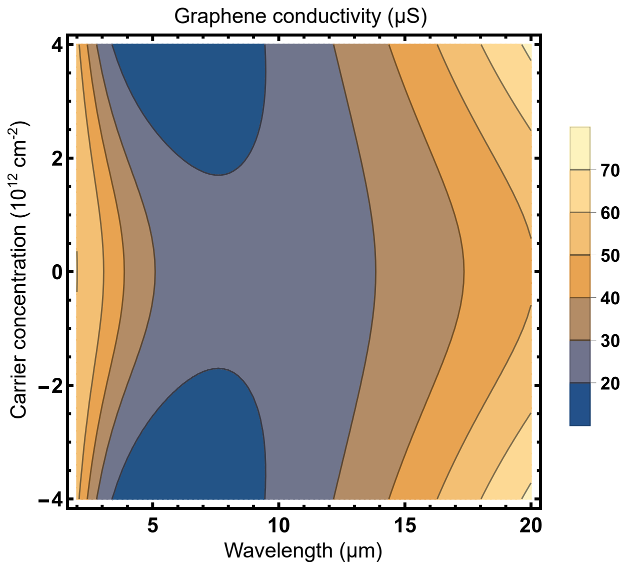


**Supplementary Figure 7: Optical conductivity wavelength dependence.** Contour plot of real part of graphene's infrared optical conductivity as a function wavelength and mean carrier concentration, assuming a carrier concentration spread of $\sigma_n = 2.0 \times 10^{12}\,\mathrm{cm}^{-2}$ and a Drude scattering time of $\tau_{IR} = 40\,\mathrm{fs}$.

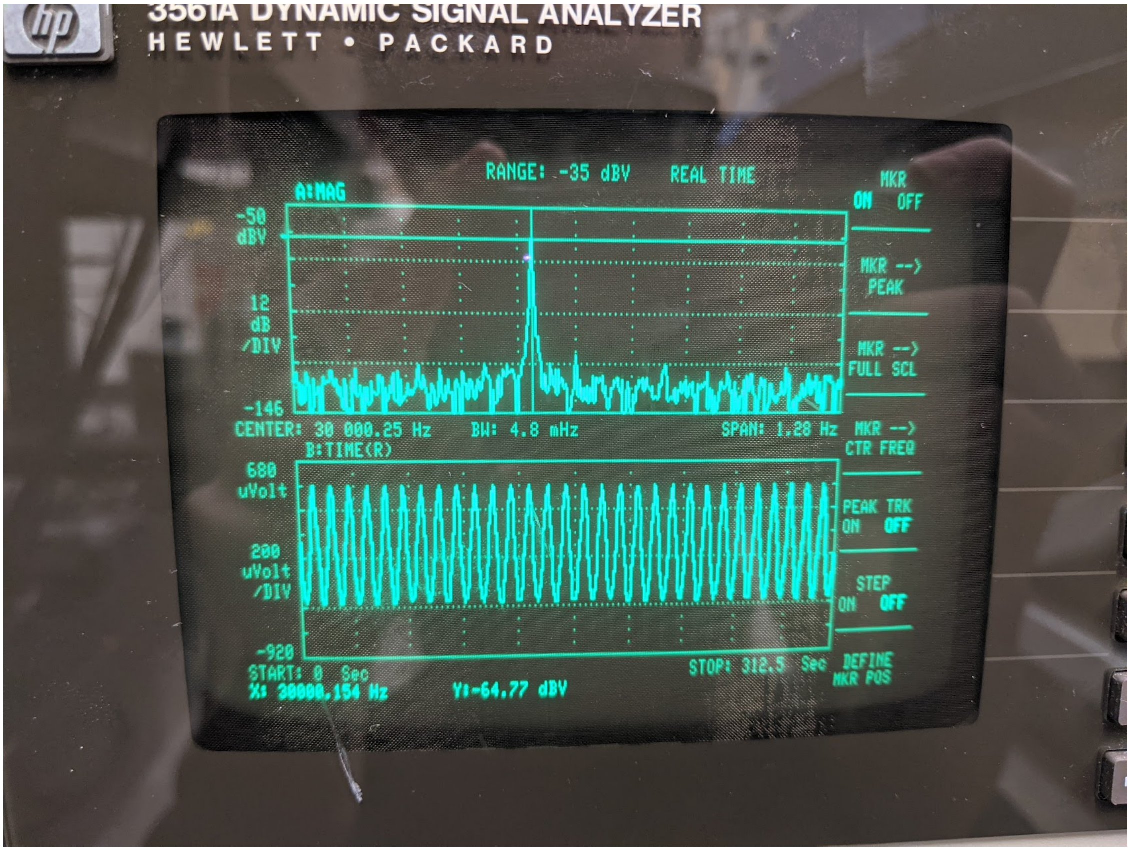


**Supplementary Figure 8: Long-term responsivity stability.** The spike in the frequency content of the photovoltage produced by illuminating the device with light modulated at 30 kHz falls below the noise floor at an offset frequency of less than 0.1 Hz, indicating long-term responsivity stability. The acquisition time for this trace was 312.5 seconds.

## Supplementary References

[1] Lee, B. H., Kang, L., Nieh, R., Qi, W.-J. & Lee, J. C. Thermal stability and electrical characteristics of ultrathin hafnium oxide gate dielectric reoxidized with rapid thermal annealing. *Applied Physics Letters* **76**, 1926–1928 (2000).

[2] Robertson, J. High dielectric constant oxides. *European Physics Journal-Applied Physics* **28**, 265–291 (2004).

[3] Siebert, R. & Müller, J. Infrared integrated optical evanescent field sensor for gas analysis: Part i: System design. *Sensors and Actuators A: Physical* **119**, 138–149 (2005).

[4] Snyder, A. W. & Love, J. D. *Optical waveguide theory* (Chapman and Hall, London, 1983), first edn.

[5] Visser, T. D., Blok, H., Demeulenaere, B. & Lenstra, D. Confinement factors and gain in optical amplifiers. *IEEE Journal of Quantum Electronics* **33**, 1763–1766 (1997).
